# Development of Lignin-Containing Cellulose Nanofibrils Coated Paper-Based Filters for Effective Oil-Water Separation

**DOI:** 10.3390/membranes13010001

**Published:** 2022-12-20

**Authors:** Anna Mittag, Md Musfiqur Rahman, Islam Hafez, Mehdi Tajvidi

**Affiliations:** 1 Department of Chemical and Biomolecular Engineering, University of Notre Dame, Notre Dame, IN 46556, USA; 2 Laboratory of Renewable Nanomaterials, School of Forest Resources, University of Maine, 5755 Nutting Hall, Orono, ME 04469, USA

**Keywords:** lignin-containing cellulose nanofibrils, oil-water separation, water filtration, surface modification

## Abstract

New methods of oil-water separation are needed as industrialization has increased the prevalence of oil-water mixtures on Earth. As an abundant and renewable resource with high oxygen and grease barrier properties, mechanically refined cellulose nanofibrils (CNFs) may have promising applications for oil-water separations. The unbleached form of these nanofibrils, lignin-containing CNFs (LCNFs), have also been found to display extraordinary barrier properties and are more environmentally friendly and cost-effective than CNFs. Herein, both wet and dry LCNF-modified filter papers have been developed by coating commercial filter paper with an LCNF suspension utilizing vacuum filtration. The LCNF-modified filters were tested for effectiveness in separating oil-water emulsions, and a positive relationship was discovered between a filter’s LCNF coat weight and its oil collection capabilities. The filtration time was also analyzed for various coat weights, revealing a trend of high flux for low LCNF coat weights giving-way-to predictions of a coat weight upper limit. Additionally, it was found that wet filters tend to have higher flux values and oil separation efficiency values than dry filters of the same LCNF coat weight. Results confirm that the addition of LCNF to commercial filter papers has the potential to be used in oil-water separation.

## 1. Introduction

Due to rapid industrial and economic development, the need for oil and hence the prevalence of oil-water mixtures has increased dramatically in recent years. These mixtures have many damaging effects, however, threatening human health, disrupting ecosystems and the environment, and wasting valuable resources [1]. Specifically, oily wastewater pollution affects groundwater and drinking water, endangers human health, affects crop production, destructs the natural landscape, and contributes to atmospheric pollution [2]. With the international need for oil consistently increasing, it is likely that these issues of oily wastewater pollution will only compound in future years. This is incredibly problematic as it limits the amount of usable, clean water—an essential resource that is already scarce in many parts of the world. By the 2000s, 58% of the global population lived under some level of water scarcity and that number is only projected to increase [3]; hence methods of oil-water separation are crucial for the health of humans and the environment. Specifically, techniques of oil-water emulsion separation need to be improved as these mixtures are thermodynamically stable and therefore difficult to manipulate [1]. Current methods of separation include gravity separation, chemical dispersants, centrifugation, and flotation—all high energy consuming, costly, complex, and possibly polluting processes [4] and thus an energy-conscious, eco-friendly, and low-cost solution is needed.

One promising solution may be to incorporate cellulose nanofibrils (CNFs) into paper-based filters. Being nontoxic and renewable, paper is a desirable material to work with [5]. Traditional filtration approaches, however, have limited separation efficiency for stable emulsified oil-water mixtures [6]; due to their large pore size and limited wettability, commercial filter papers are not successful in effectively separating oil-water emulsions [4]. Recent research on CNFs has identified impressive oxygen and grease barrier properties that may allow us to functionalize the filter paper for use in these applications. Cellulose is advantageous due to its renewable nature, biodegradability, low cost, and nontoxicity [7], and it can be extracted from wood, plants, algae, bacteria, and even tunicates, a family of sea animals [8]. Cellulose is a linear homopolysaccharide linked by *β* 1–4 glucosidic bonds, with the molecular formula of (C_6_H_10_O_5_)_n_ [9]. By applying mechanical treatments such as grinding, cryocrushing, or microfluidization to both the amorphous and crystalline regions of cellulose, CNFs can be generated [8].

Recent work has found that CNFs can help produce films with increased barrier properties against oxygen and grease [10] due largely to the hydrogen bonding between hydroxyl groups [11] that forms a tight impermeable layered structure. While most literature on cellulose nanomaterials has focused on bleached nanofibrils, it is also possible to produce nanofibrils from unbleached fibers as well as recycled cardboard. These resulting nanofibrils are known as lignin-containing cellulose nanofibrils (LCNFs) and are produced from unbleached chemical pulps, thermo-mechanical pulps, or old corrugated containers (OCC) [12]. LCNFs offer many of the same advantages as CNFs but additionally have a lower production cost and environmental impact for a higher yield [13]. In fact, a previous study reported that producing 1 kg of LCNFs is 100 times cheaper than producing 1 kg of TEMPO-oxidized CNFs, a cellulose nanomaterial widely utilized in the literature [12]. There is also promising evidence that LCNF-based materials may even be superior to CNF-based materials in some applications. In recent experiments, LCNF-modified packaging has displayed excellent oil barrier properties, outperforming CNF-modified packaging, likely because of LCNF’s lower polarity and surface energy [5]. Keeping all this in mind, it would be of great value to utilize LCNFs in oil-water separation as this implementation would have multi-faceted benefits.

Previous work that investigated self-standing CNF and LCNF films revealed their production is expected to be slow and energy-consuming [14]; hence an approach that modifies currently available commercial filter paper was taken in this study. One previously used method for applying CNFs and LCNFs to materials was to coat a surface and then thermally dry it [15,16]. Another study at Wuhan University [4] utilized tunicate cellulose nanocrystals to coat filter papers through physical and chemical (i.e., using a crosslinking agent) methods. Filters prepared via chemical crosslinking exhibited better oil separation than those prepared via physical modification. More recent studies have shown cellulose nanofibrils to be sturdier and easier to handle than nanocrystals [17], so this may be beneficial in regard to the reusability of our developed filters. Recently, other studies have found promising water-filtration results utilizing membranes made from natural resources. These include membranes formed out of polylactic acid and gelatin [18], chitosan-cellulose nanocrystals [19], date seed biomass [20], graphene oxide, sodium alginate, and lignin [21]. Other attempts involved incorporating hydrophobic polymers such as poly(perfluorooctylethyl methacrylate) or poly(methylhydrosiloxane) with cellulosic materials [22,23]. However, these treatments are often non-sustainable and may raise health concerns depending on the chemicals used in these polymeric materials. Despite the successful attempts, there is still a need to explore and provide a proof of concept of low-cost alternatives for oil/water separation.

The overarching goal of this study was to contribute knowledge to advance the development of an eco-friendly and low-cost filter using LCNFs for effective and efficient oil-water separation for use in oil-spill accidents and oily wastewater environments. In this work, a vacuum filtration technique was utilized to apply uniform layers of LCNFs to the filter paper without crosslinking agents or thermal drying, helping to minimize energy consumption and provide a low-cost and biodegradable option for oil filters. This approach was modeled after a previous study at the University of Maine [5] but differs from it by using filter paper as a starting material and by producing it for oil-water separation rather than packaging. Our objectives were to improve separation performance and optimize the time efficiency of paper filters through physical modification using LCNFs.

## 2. Materials and Methods

### 2.1. Materials

Whatman grade 5 filter paper (10 cm diameter and 2.5 µm pore size) was purchased and utilized throughout experiments, both as a control sample and as the base for LCNF-modified filters. LCNFs were obtained from the University of Maine’s Process Development Center (PDC) and were made by mechanically refining old corrugated containers (OCC). The constituents of the OCC LCNFs were 61.86% cellulose, 18.05% hemicelluloses, and 16.67% lignin [24]. The as-received LCNF contained 2 wt% solids but was diluted to 0.1 wt% solids prior to use. For the oil/water emulsion, vegetable oil was purchased from the local grocery store, analytical grade Tween80 surfactant was obtained from MilliporeSigma (Burlington, MA, USA), and analytical grade red oil O (C_26_H_24_N_4_O) dye was sourced from Alfa Aesar (Haverhill, MA, USA).

### 2.2. Preparation of LCNF-Modified Filter Papers

**Figure 1:**
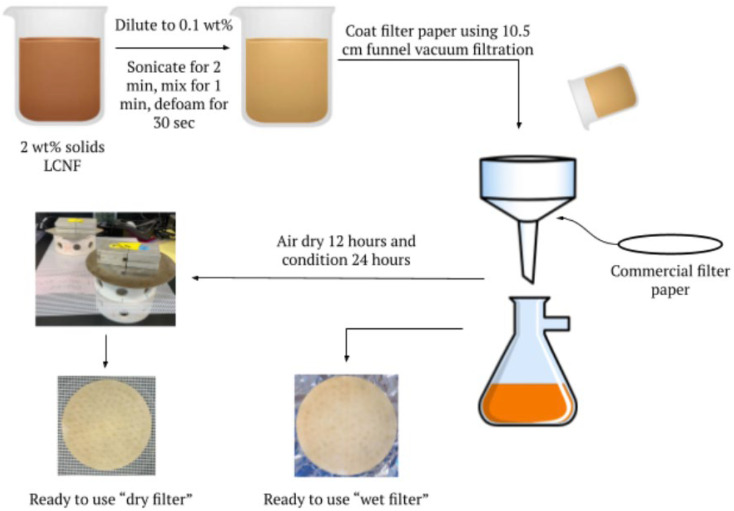
Summary of filter coating process of both wet and dry LCNF-modified filter papers.

The first step in preparing wet and dry LCNF-modified filter papers was to produce an LCNF suspension. The LCNF suspension was prepared by diluting the as-received LCNFs to 0.1 wt% solids, sonicating the 0.1 wt% slurry for 3 min at 90% duty cycle and output control value 3 (Branson 450 Sonifier, Ultrasonics Corporation, Danbury, CT, USA), and then agitating the mixture using a planetary centrifugal mixer (Thinky 310, Thinky Corporation, Tokyo, Japan) by mixing for 1 min at 2200 rpm and then defoaming for 30 s at 2000 rpm. The next step was to deposit the LCNF suspension onto the filter’s surface. After testing a multitude of coating methods, it was determined that the most effective way to evenly coat the filters with LCNF was to use vacuum filtration. To do so, a commercial filter paper was placed into a Buchner funnel (10.5 cm diameter) and coated with water to adhere to the funnel, then the LCNF suspension was poured on top of the filter using a glass stirring rod to ensure even distribution, the vacuum filtration was run at 20 inHg until the water in the suspension had successfully passed through the filter and the LCNFs were left on top. Multiple amounts of the 0.1 wt% LCNF suspension were utilized to create different coat weights on the filters. After removing the LCNF-modified filters from the funnel, they were either used immediately in oil-water separation testing (for the wet filters) or air-dried (for the dry filters). To make sure the dry filters remained flat while drying, they were restrained and weighed down by PVC rings and metal weights. Figure 1 displays a schematic of the filter modification process. Both wet and dry filters with LCNF coat weights spanning from 0 g per square meter (gm^−2^) to 9 gm^−2^ were created and utilized in testing.

### 2.3. Filter Characterization

The dry LCNF-modified filter paper coat weights were calculated using the mass differences of the filters before alterations and after the LCNF coating had dried. The wet LCNF-modified filter paper coat weights were calculated using the mass of LCNFs suspension that was applied to the commercial filters. In both cases, results were reported in grams of LCNF per square meter of filter paper (gm^−2^).

The morphology of the LCNF-modified filter papers was evaluated through scanning electron microscopy (SEM) imaging. A Zeiss Nvision 40 scanning electron microscope (SEM; Oberkochen, Germany) machine was utilized to perform SEM imaging on an unmodified control filter as well as several dry LCNF-modified filters with varying coat weights. To prepare filters for SEM imaging, samples were cut using a sharp blade so that they could fit on the SEM sample stub using a double-sided carbon tape followed by a 4 nm of sputter coating of Au/Pd. SEM images were obtained at multiple magnifications. An electron high tension (EHT) voltage of 3 kV was maintained at the time of scanning. Only dry LCNF-modified filters could be visualized by SEM imaging as this SEM could not handle the moist nature of the wet LCNF-modified filters.

To determine the wettability of modified filter papers, a Krüss mobile surface analyzer (Krüss GmbH, Hamburg, Germany) was used to measure the contact angle of two drops of liquids—polar water and non-polar diiodomethane. Each drop was approximately 1 μL in volume and the contact angles were measured after 1 s of the drop being on the surface. After measuring the contact angles for each LCNF coat weight, the surface free energy (SFE) and its polar and disperse components were calculated using the Owens, Wendt, Rabel, and Kaelble (OWRK) model [25].

### 2.4. Preparation of Oil-Water Emulsions

Oil-in-water emulsions were prepared using a 1:99 (oil:water) mass ratio. To create a stable oil-water emulsion, a surfactant was needed. Surfactants are classified by hydrophilic-lipophilic balance (HLB), which is described as a numeric value that conveys the balance of the size and strength of two opposite groups, hydrophobic and lipophilic groups, in an emulsifier [26]. To create a stable oil-in-water emulsion, an HLB between 8 and 18 is needed. In our experiments, Polysorbate 80, a non-ionic surfactant commonly known as Tween80, was utilized. Tween80 has an HLB of 15 and thus was an ideal emulsifier for our experiment. For an 80 mL emulsion, 0.16 g of Tween80 was used. Additionally, red oil dye was utilized to help visualize results. The oil, water, surfactant, and dye were mixed for 1 min at 2200 rpm and then defoamed for 30 s at 2000 rpm in the planetary centrifugal mixer (Thinky 310, Thinky Corporation, Tokyo, Japan) to effectively prepare a homogenous emulsion. Using optical microscopy, the emulsion particle size was measured—on average oil particles were 6.8 microns in diameter—emulsions were determined to be stable if they did not separate after 15 min.

### 2.5. Oil-Water Separation Process

**Figure 2:**
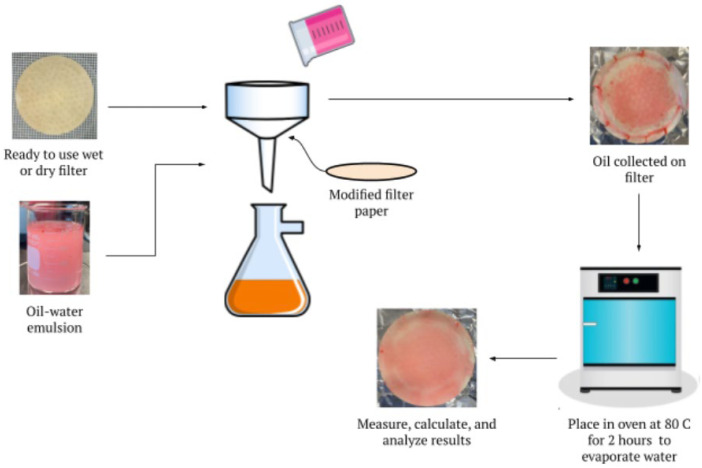
Summary of oil-water separation process using vacuum filtration.

The oil-water separation tests were carried out on a vacuum filtration setup, using a 9.5 cm diameter funnel so that the filters formed a cup-like shape, ultimately preventing liquid from bypassing the filter. A total of 80 mL of the oil-water emulsion was poured over the LCNF-modified filter papers using a glass stir rod to ensure even distribution, and the vacuum filtration was performed under a pressure of 27 inHg. A container was used to collect the filtrate, as ideally the water passed through the filter and the oil was collected by the filter. The amount of filtrate collected was measured to compare with the initial quantity of liquid deposited onto the filter. Additionally, the time of filtration was recorded, with the filtration being considered complete when the time between two consecutive drops surpassed 10 s. After filtration, the used filter was placed in an oven at 80 °C for 2 h to allow the excess water to evaporate while still retaining the oil collected. These conditions proved to be an ample amount of time and a high enough temperature to evaporate the water in our experiments. After drying, the collected oil mass was calculated gravimetrically by subtracting the initial dry weight of the filter from its dry weight after filtration. A schematic representation of this oil-water separation procedure is displayed in Figure 2.

Various equations were utilized in order to compare useful variables between the filters tested. The water flux, J (L m^−2^ h^−1^ bar^−1^), was calculated using Equation (1):

J = V/(At∆P),(1)

where V (L) is the permeated water volume, A (m^2^) is the surface area of the funnel, t (h) is the drain time, and ΔP (bar) is the pressure across the filter paper.

The oil separation efficiency, R_1_ (%), was calculated with Equation (2):

R_1_ = (m_oil f_/m_oil i_) × 100% (2)

in which m_oil f_ (g) and m_oil i_ (g) represented the mass of oil in the filtrate and the initial mass of oil used in the emulsion, respectively.

The water separation efficiency, R_2_ (%), was calculated using Equation (3):

R_2_ = (m_water f_/m_water i_) × 100% (3)

where m_water f_ (g) was the mass of water after the separation process and m_water i_ (g) was the mass of water before the separation process.

Optical microscopy was also utilized in order to visualize oil droplets in the emulsions before and after filtration through the various filters. Image J software (U.S. National Institutes of Health, Bethesda, ML, USA) was used to estimate the size of the droplets.

## 3. Results and Discussion

### 3.1. Filtration Outcomes

**Figure 3:**
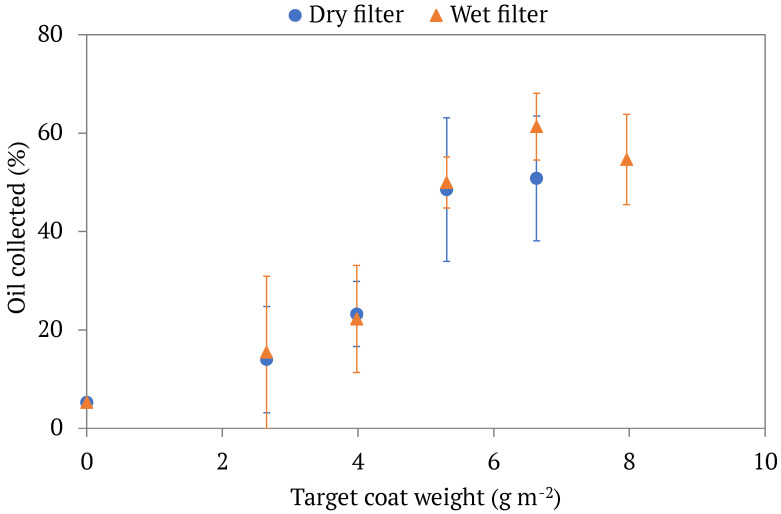
The oil separation efficiencies, calculated by Equation (2), of different LCNF-modified filters by coat weight. Both dry and wet filters are displayed. Each point represents the average oil separation efficiency of multiple trials of modified filters with the same target coat weight, with the standard deviations of said values being displayed in the error bars.

As the LCNF coat weight increased on filter papers, so did the oil separation efficiencies, R_1_. In other words, the more LCNF that was deposited on a filter, up to a coat weight of around 6.6 gm^−2^ gsm, the more oil was collected by it in the filtration process. At approximately 6.6 gm^−2^, however, the increase in oil separation efficiencies seems to level off a bit. Figure 3 displays an oil collection graph in which the x-axis represents a filter’s LCNF coat weight, and the y-axis represents the oil collection efficiency as calculated in Equation (2). As seen in the graph, an unmodified commercial filter could collect approximately 5% of the oil contained within an emulsion, while the LCNF-modified filters produced in this study collected up to 61% of the oil. This is due to the superior oil barrier properties of LCNFs that previous studies have identified [5]. While the mechanism for these properties is not well known, it is possible that lignin adds water resistance and crack-fold resistance. To further investigate the role of lignin, a control sample of dry CNF-modified filter paper was tested (coat weight: 7.5 gm^−2^).. At 7.5 gm^−2^, the collected oil percent of the CNF-based filter was 75%, whereas that of the LCNF-modified filter was 51% at 6.6 gm^−2^. The key difference between LCNF and CNF layers used in modifying the filter paper is the greater extent of hydrogen bonding within the CNF film as opposed to LCNF film, possibly resulting in a less porous coating layer. This result indicates the need for a tight network of micro- and nano-sized fibers to achieve a favorable oil separation. Based on this comparison, the role of lignin as a factor in the separation process is not clear. However, using LCNF-based materials instead of CNF-based materials is still favorable as they require less energy to produce.

As explained in the Methods section, the filters were prepared via dry and wet approaches. It was hypothesized that a wet filter could result in a better oil separation if nano-sized fibrils were collected on the fibers of filter paper, hence resulting in better separation efficiency. However, from Figure 3, wet LCNF-modified filters had comparable oil separation efficiencies, R_1_, to dry LCNF-modified filters. Furthermore, it is worth noting that a re-wetted dry filter is not the same as a wet filter as during the drying process, shrinkage occurs and layers of LCNFs dry together, resulting in decreased swelling abilities when re-wetted (also known as hornification) [27]. Based on these findings, it is evident that the application of a uniform and tightly packed LCNF layer on the filter paper enabled the rejection of oil from the oil-water emulsion. However, further experiments are needed to verify whether or not adsorption contributes to the separation mechanism.

**Figure 4:**
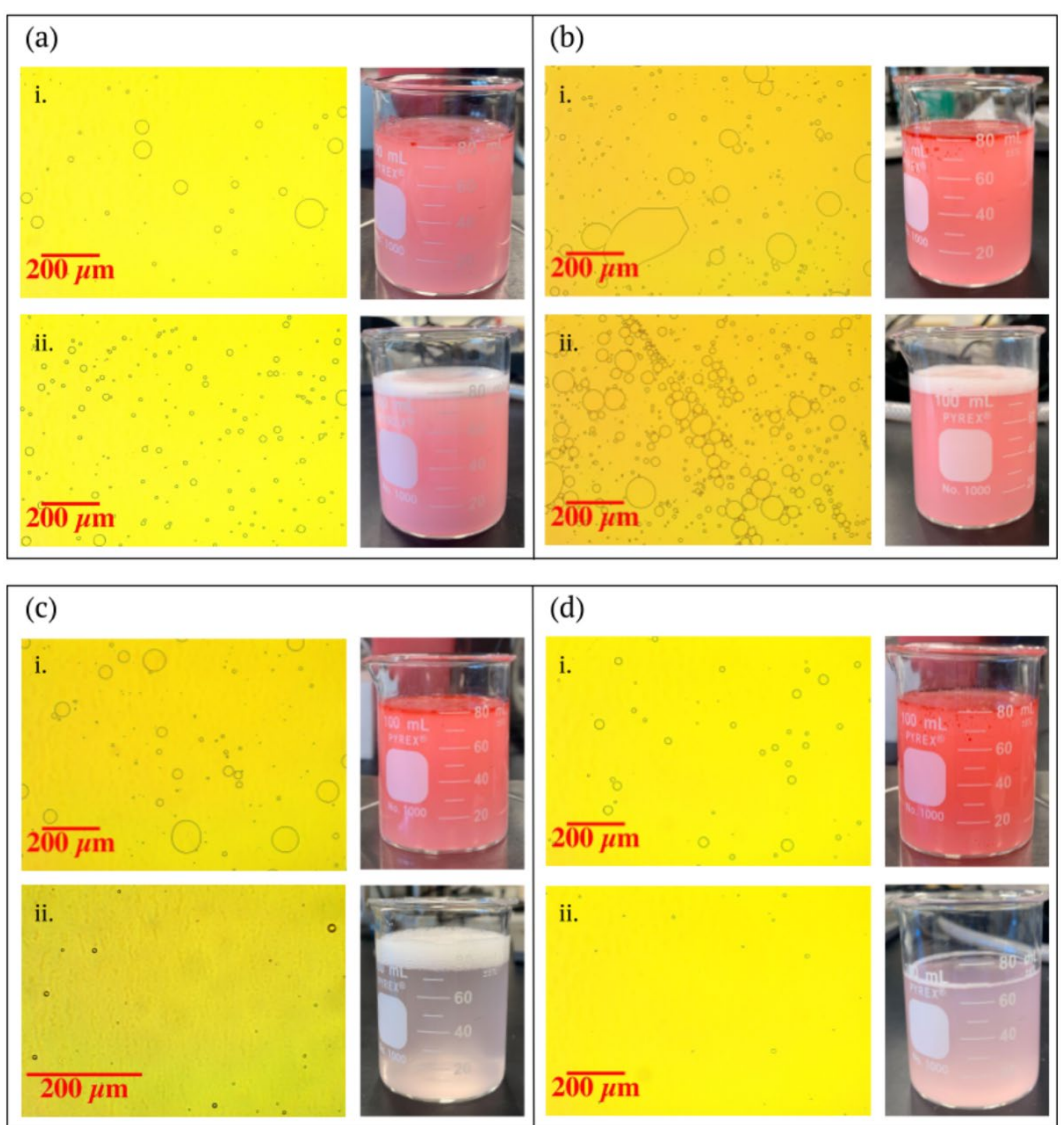
Photographs and microscopy imaging of oil-water emulsions before (i) and after (ii) filtration through an (**a**) unmodified control filter, (**b**) wet 2.44 gm^−2^ filter, (**c**) wet 8.34 gm^−2^ filter, and (**d**) dry 8.22 gm^−2^ filter.

The visual results of the separation experiments also confirmed these findings. Figure 4 shows the oil-water emulsions before and after filtration through a variety of wet and dry LCNF-modified filters, as well as through an unmodified control filter paper. Both photos of the emulsions as well as optical microscopy images of the particles are included for the unmodified filter and three LCNF-modified filters (wet 2.44 gm^−2^, wet 8.34 gm^−2^, and dry 8.22 gm^−2^). The oil in the emulsions was dyed red so that its presence could be clearly seen in the filtration process. It is apparent that the unmodified filter paper (Figure 4a) did a poor job removing oil from the emulsion, as seen in the high prevalence of red dye in the filtrate image and in the high number of large oil droplets in the post-filtration microscopy imaging. The low coat weight wet LCNF-modified filter (Figure 4b) also performed rather poorly, displaying visual results comparable to the unmodified filter—with the filtrate color and the post-filtration oil particle sizes being relatively similar. The two high coat weight LCNF-modified filters (Figure 4c,d) show the large effect of LCNFs on oil collection ability. The post-filtration photos in both these cases are much clearer than the previous examples, showing very little red dye, and the post-filtration microscopy images show much smaller oil droplets. Between these two filters, which have relatively the same coat weight, it is apparent that the wet LCNF-modified filter (Figure 4c) is more effective than the dry LCNF-modified filter (Figure 4d) in removing oil, as seen by the clearer filtrate and smaller oil particles of Figure 4c.

The use of microscopy imaging to analyze the size and morphology of oil droplets was applied to a number of other LCNF-modified filters in order to further explore the effects of both coat weight and wet versus dry filter conditions. Table 1 displays the post-filtration average oil particle size (calculated using Image J) and coefficient of variation of these values for several wet and dry LCNF-modified filters, as well as an unmodified control filter. Few clear conclusions could be made from the values collected through microscopy images as a significant pattern did not emerge. For instance, the average oil particle size post-filtration using an unmodified filter was 8.4 µm, and using a dry LCNF-modified filter of coat weight 7.2 gm^−2^ it was nearly identical at 8.1 µm. Additionally, it may be possible that smaller droplets coalesce after passing through the filter to make larger droplets, hence increasing the average particle size seen through microscopy. One trend that was evident through microscopy images, however, was that dry LCNF-modified filters had a much lower coefficient of variation (ranging from 12.9–94.9%) than the wet LCNF-modified filters (ranging from 73.1–660.9%). The lower variation of the oil particles’ sizes after passing through the dry LCNF-modified filters may be due to the LCNF coat layer being more uniform after drying. When re-wetting dry filters during filtration, the fibers have a decreased ability to swell [27], rendering them more uniform than never-dried filters.

**Figure 5:**
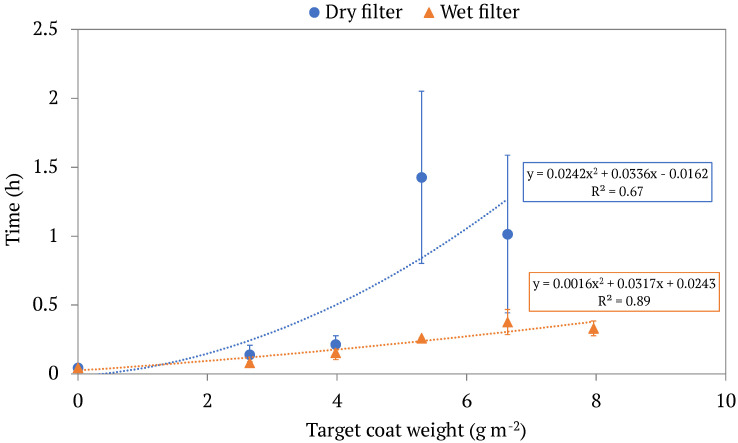
The filtration time of different LCNF-modified filters by coat weight. Both dry and wet filters are displayed, each set with its own respective trendline. Each point represents the average filtration time of multiple trials of modified filters with the same target coat weight, with the standard deviations of said values being displayed in the error bars.

While oil collection is of utmost importance in the development of our filters for oil-water separation, it is crucial to balance collection effectiveness with time efficiency so that the filters are usable in the real world. As expected, filters with a higher coat weight of LCNFs had a longer filtration time, t. Figure 5 shows this trend, displaying filtration time curves in which the x-axis represents a filter’s LCNF coat weight, and the y-axis represents the time to filter 80 mL of an emulsion through said filter. Trendlines for both the wet and dry LCNF-modified filters exhibit positive slopes, illustrating that filtration time increases greatly with increased LCNF coat weights. Another finding from our study was that for filters with similar coat weights, wet LCNF-modified filters had a shorter filtration time, t, than dry LCNF-modified filters. One possible explanation for this finding is that as the filter dries, the space between layers of LCNF decreases, and thus there is less free space for water to travel, so there is simply more liquid trying to go through a tighter space.

**Figure 6:**
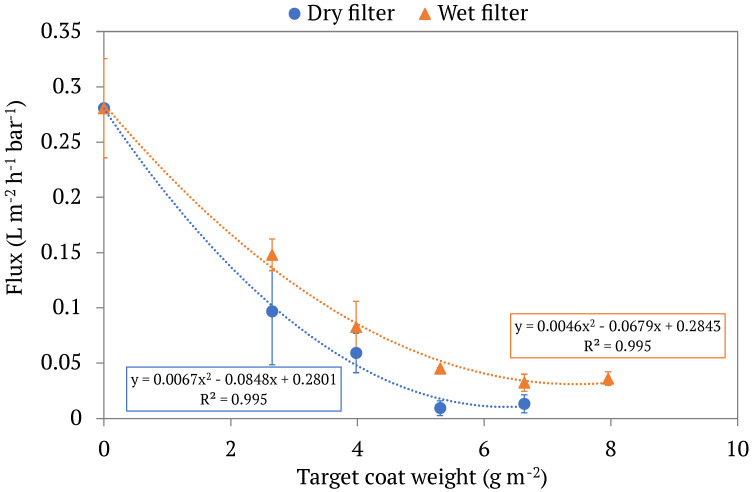
The flux values, calculated by Equation (1), of different LCNF-modified filters by coat weight. Both dry and wet filters are displayed, each set with its own respective trendline. Each point represents the average flux of multiple trials of modified filters with the same target coat weight, with the standard deviations of said values being displayed in the error bars.

With the efficiency of time and energy in mind, it is critical to determine an upper limit coat weight, i.e., the maximum amount of LCNF that can be applied to a filter without massively inhibiting its ability to allow water to pass through. To aid in this analysis, we calculated flux values, J, of each filter tested using Equation (1). Figure 6 displays a graph of the filters’ flux values in relation to their coat weight. Filters with a higher coat weight displayed a lower flux than filters with a low coat weight, and dry LCNF-modified filters displayed a lower flux (on average) than wet LCNF-modified filters. By incorporating trendlines into the flux graphs, we are able to visualize the coat weight at which water flow levels off, helping to predict the maximum coat weight of LCNF that one could apply to a filter paper in both wet and dry conditions. This is an important aspect of the study as it is the balance between oil collection efficiency (which increases with coat weight) and flux (which decreases with coat weight) that will enable a filter to be both effective and realistically usable. The flux of both dry and wet LCNF-modified filters appeared to level off around a coat weight of 5.3 gm^−2^. While previous works have not investigated the flux of filter papers coated in OCC LCNFs, there have been studies that utilize tunicate cellulose nanocrystals (TCNCs) and bamboo-based LCNFs in similar applications [4,28]. Flux rates are much higher in each of these studies (reaching up to 317.7 L m^−2^ h^−1^ bar^−1^) than the values found in this experiment, but this is likely because both the TCNCs and the bamboo-based LCNFs are able to coat the inside of the filter paper pores rather than just coating the surface of the paper as we are. While using OCC LCNF to coat commercial filter paper means our fluxes are much lower than these reported values, it also means the process of modifying filters is simpler and more cost-effective.

**Figure 7:**
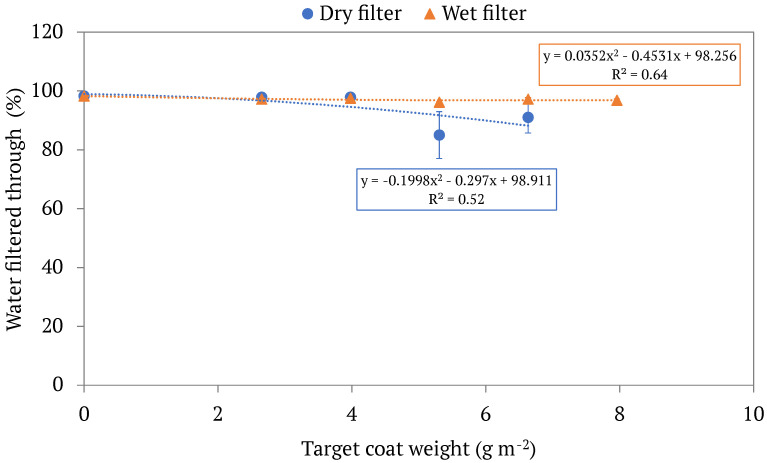
The water separation efficiency values, calculated by Equation (3), of different LCNF-modified filters by coat weight. Both dry and wet filters are displayed, each set with its own respective trendline. Each point represents the average water separation efficiency of multiple trials of modified filters with the same target coat weight, with the standard deviations of said values being displayed in the error bars.

Another important factor to consider regarding our filters’ usability and efficiency is what we call the water separation efficiency, R_2_, as noted in Equation (3). Water separation efficiency is defined as the percent of water originally in the emulsion that is recovered after the filtration process. A high water separation efficiency means that most of the water was able to pass through the filter, while a low value would signify that some water was collected with the oil (ultimately wasting it). The water separation efficiencies, R_2_, for various filters are displayed in Figure 7. The unmodified control filters had water separation efficiencies around 98%, meaning that even in uncoated filters approximately 2% of the water contained in the emulsion was lost. The water separation efficiencies of wet LCNF-modified filters remained relatively constant even as coat weight increased (R_2_ values ranged from 95.98–98.29%), while those of dry LCNF-modified filters decreased as coat weight increased (with R_2_ values below 90% for all of the higher coat weights). This means that the wet LCNF-modified filters waste less water in the separation process, a finding once again is contributed to the smaller pores and more densely packed layers characteristic of dry LCNF-modified filters.

### 3.2. Filter Properties

**Figure 8:**
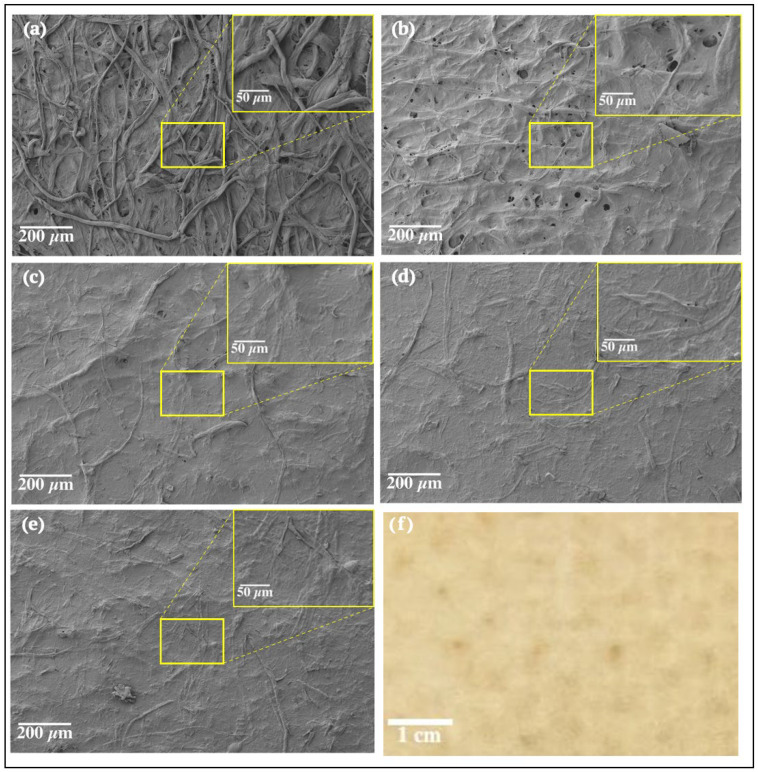
Surface SEM images of dry LCNF-modified filters at various coat weights: (**a**) uncoated control filter, (**b**) 3.11 gm^−2^, (**c**) 5.49 gm^−2^, (**d**) 6.02 gm^−2^, and (**e**) 9.54 gm^−2^ at different magnifications (85× and 500×). (**f**) Magnified image of dry filter paper.

SEM images of the surface of an unmodified filter and of dry LCNF-modified filters of various coat weights were captured and are displayed in Figure 8. The commercially available filter paper was composed of heterogeneous microfibers with distinct borders. SEM imaging and analysis clearly showed the entangled network of individual fibers within the unmodified filter paper (Figure 8a). The LCNF-modified filter papers, on the other hand, displayed a more dense and uniform morphology due to the formation of a tight LCNF layer on the surface. SEM images of the LCNF-modified filters show a much smoother filter surface (Figure 8b–e) than the control filter (Figure 8a). As the LCNF coat weight of filters increased, the size of the pores in the filters visibly decreased and the presence and entanglement of LCNFs surrounding filter paper fibers increased. While the filter lightly coated in LCNF (3.11 gm^−2^) still had a number of voids visible in the 85× magnified images (Figure 8b), the filter most heavily coated in LCNF (9.54 gm^−2^) had no visible voids (Figure 8e), even at the 500× magnification we utilized. The tight network created by high coat weights of LCNF is believed to be one of the reasons responsible for creating barrier properties against a number of substances [29]. While SEM imaging could only be performed on the dry LCNF-modified filters, it is assumed that the wet LCNF-modified filters had similar trends in decreasing pore size with increasing LCNF coat weights. One potential difference, however, between the morphologies of the dry and wet LCNF-modified filters could be that wet filters are packed comparatively less tightly. This assumption is due to the fact that LCNF experiences shrinkage after drying, and therefore it is likely that the pores of dry LCNF-modified filters would be slightly smaller than the pores of wet LCNF-modified filters of relatively similar coat weights.

Surface free energies (SFE) were calculated in order to evaluate the barrier properties of the various filters, as SFE has a great influence on a material’s wetting and adsorption of water, oil, and grease [13]. Previous literature has shown that LCNF promotes a larger water contact angle and lower surface energy than CNF due to lignin creating greater water repellency [5]. Table 2 summarizes the water contact angle, diiodomethane contact angle, surface free energy, dispersive free energy, and polar surface free energy of filters coated in various weights of LCNF. Once again, only dry LCNF-modified filters were characterized due to the limitations of the Krüss mobile surface analyzer. Values for the unmodified commercial filters could not be measured either due to these filters’ extreme porosity or high hydrophilicity. Our findings show that filters with higher LCNF coat weights tend to have lower SFE values. These SFE values (which range from 39.5 mN m^−1^ to 62.8 mN m^−1^) are comparable with those reported in the literature, with a prior study finding SFEs of LCNF-coated materials ranging from (43.6–62.48 mN m^−1^) [30]. The SFE of our filter with a low LCNF coat weight was much higher than our other filters’ SFE values. This is likely due to the fact that our lightly coated filter had an LCNF coat weight of 2.87 gm^−2^, which as seen in previous sections, leaves many free pores in the filter surface, while our other filters have an LCNF coat weight between 5.49–9.23 gm^−2^.

## 4. Conclusions

Coating commercial filter papers with LCNFs can improve oil-water separation capabilities. Wet LCNF-modified filters collected up to 61% of oil, while dry LCNF-modified filters collected up to 51% of oil in experiments. Both of these modification techniques resulted in oil collection improvement, however, as unmodified filters only collected up to 5% of the oil. Wet LCNF-modified filters exhibited a higher flux than dry LCNF-modified filters, allowing for more time- and energy-efficient processes. Water waste was also lower when wet LCNF-modified filters were used compared to dry LCNF-modified filters, with water separation efficiency values above 95% for wet LCNF-modified filters but some water separation efficiency values falling below 90% for the dry LCNF-modified filters. Increasing the LCNF coat weight increased the oil collection in both the wet LCNF-modified and dry LCNF-modified filters. At the same time, however, flux decreased as LCNF coat weight increased. From SEM images, we can see both these trends are caused by the smaller pores created by densely packed and entangled lignin-containing cellulose nanofibrils in highly coated films. A surface analysis of the modified filters showed that filters more densely coated with LCNF displayed lower surface free energies than lightly coated filters, 9.23 gm^−2^ filters had SFEs of 39.5 mN m^−1,^ while 2.87 gm^−2^ filters had SFEs of 62.8 mN m^−1^, which also helps to explain the findings of this study. The modification techniques described in this work are low-cost, readily available, easily replicable, and energy-efficient, thus showing promise for a broader impact. Given well-established platforms for coating with CNF-based materials, there is potential for scale-up applications, but that is not within the scope of this project. Additionally, the filters and LCNFs are biodegradable and thus can decompose naturally without leaving a larger footprint. Ultimately, this method of modifying commercial filter papers with LCNFs can help produce a filter that is more economical, environmentally friendly, and attainable than many other oil-water filtration technologies. Future work will involve collecting real-time data for an extended period of time to gain further insights into the mechanism of separation.

## Figures and Tables

**Table 1:** The average size of oil particles in filtrates after running an oil-water emulsion through wet and dry filters at various LNCF coat weights.

Wet Filters			Dry Filters		
Coat Weight (gm^−2^)	Average Oil Particle Size (µm)	Coefficient of Variation (%)	Coat Weight (gm^−2^)	Average Oil Particle Size (µm)	Coefficient of Variation (%)
0	8.4	73.1	0	8.4	73.1
2.44	7.0	158.0	7.16	8.1	94.9
3.79	2.3	660.9	8.22	4.7	74.8
7.49	5.5	181.6	8.76	2.8	60.0
8.34	1.0	170.3	10.62	2.7	12.9

**Table 2:** Water contact angles, diiodomethane contact angles, and surface free energy and its components for dry LCNF-modified filters at various coat weights.

Coat Weight (gm^−2^)	2.87	5.49	6.02	9.23
Water contact angle (°)	49.9 (±11.4)	78.5 (±6.1)	81.1 (±5.7)	84.9 (±4.4)
Diiodomethane contact angle (°)	19.2 (±5.4)	46.1 (±3.1)	43.0 (±4.5)	45.0 (±6.2)
Surface free energy (mN m^−1^)	62.8 (±7.5)	41.2 (±4.0)	41.5 (±4.3)	39.5 (±4.7)
Dispersive surface energy (mN m^−1^)	48.0 (±1.5)	36.4 (±1.7)	38.1 (±2.4)	37.0 (±3.3)
Polar surface energy (mN m^−1^)	14.8 (±6.0)	4.7 (±2.4)	3.4 (±1.9)	2.5 (±1.4)

## Data Availability

Not applicable.
